# Documents Relative to the Medical College of Ohio; and the Law Regulating the Practice of Physic in Ohio

**Published:** 1833

**Authors:** 


### II. Analytical.

29. Documents relative to the *Medical College of Ohio;* and the *Law regulating the Practice of Physic in Ohio.*

I. *Dr. Drake's Resignation.*

Cincinnati, Jan. 19th, 1832.

*To the Honorable Board of Trustees, of the Medical College of Ohio.*

Gentlemen:—Last summer, when I suggested and agreed to accept the chair of Clinical Medicine, in the Medical College of Ohio, as the only means in *my* power of effecting the union of the Medical Department of the Miami University, with the College, an object which you had originated and were anxious to accomplish, my chief motive was to prevent the interests of medical education, in Cincinnati, from being seriously injured by a protracted negotiation. At the moment of devising the professorship, I felt an apprehension, that its subjects and limits would be too restricted in the existing condition of the Hospital, to afford me an opportunity of being useful to the class; but I felt and perceived, that a state of things had been produced by others, which left me no alternative, and I accepted the chair, resolving, if my apprehensions should be realixed, to resign it at the end of the first session.

When the chair of the Institutes was declined by the gentleman, (*Dr. Pierson, of New York,*) to whom it was offered, unless he were allowed a year for preparation, and I saw that branch assigned to me, (*for the time being,*) by the unanimous vote of the Faculty, I cherished the hope, that your honorable body would not grant the favor, which he solicited; but, at once, confirm the union of the Institutes with Clinical Medicine, and by reducing the number of chairs from eight to seven, afford me a sufficient amount of duties. You have, however, preferred a different course, and I am, therefore, compelled, to request you to accept my resignation; which I wish to take effect immediately after the next public commencement.

Thirteen years have this day elapsed, since I had the honor to solicit and obtain from our Legislature, the charter of the college; and to be appointed President and Professor of the Institutes and Practice of Medicine. From that day down to the present anniversary, whether belonging to it, or to other institutions, in different and distant cities, the Medical College of Ohio, has never ceased, nor can it hereafter cease, to be with me an object of the deepest interest. Therefore it is that I send in my resignation before the end of the session, that your honorable body may be able to make and promulgate arrangements for the next course of lectures, before the class shall disperse.

In taking a final leave of an institution, which for many years, has been an object of affection, but to the prosperity of which I can no longer oontribute the influence of my feeble exertions, I cannot forego the opportunity of expressing a hope, that it will continue to flourish, until it shall reach the high distinction and impart the solid advantages anticipated in its foundation.

I have the honor to be, respectfully, your obedient servant,

DAN. DRAKE.

II. *Preamble and Resolutions of the Third District Medical Society.*

At a late meeting of the Third District Medical Society, held in Waynesville, Warren county, the following preamble and resolutions were unanimously adopted:

*Whereas,* It is believed that the legislative enactments under which we this day assemble, have for their object the improvement of medical science, we deem it not only our privilege, but our duty, to inquire into the causes which retard this improvement, and at the same time to endeavor to extend the means by which it may be elevated to its greatest eminence:

*And whereas,* Medical Schools, conducted upon liberal principles and with ability, are of the utmost importance to the advancement of this science, we have long cherished an ardent desire to witness the establishment of such an institution in Ohio:

*And whereas,* Our Legislature has bountifully provided all the pecuniary means for the founding of an institution of the character in contemplation, and the Medical College of Ohio, the recipient of this legislative beneficence, having failed to acquire a high character at home or abroad, we are constrained to ascribe this failure to the narrow policy pursued by its Board of Trustees:

*And whereas,* A system of partiality and favoritism the most extraordinary and unprecedented, has been practised by those Trustees in the appointment of a gentleman, who frankly acknowledges his incompetence, to the responsible chair of the Institutes and Medical Jurisprudence, (since abolished) for which he asked and obtained one year to qualify himself, and this too to the exclusion of accomplishments of the first order and celebrity in the United States; and there yet being talents and qualifications of the highest order, in the college, (contrary to the known wishes of the Trustees) which are so burthened by a preponderating mediocrity, that they will, ere long we fear, be compelled to seek a situation, where selfish and disgraceful partiality is not permitted to rule, regardless of the safety of society, the benefits of medical science, the policy, the reputation, and the true interest of our state; we are called upon to deprecate the abuse of power, and contribute something in support of a profession and a science long too much neglected and degraded:

*Resolved*, Therefore, that as a society, and individually, we will use all honorable means to induce our Legislature, at its next regular session, to so change or extend the Board of Trustees of the Medical College of Ohio, as that it may thereafter be governed upon such principles as shall conciliate confidence and patronage, and promote those high interests, which were labored for in its foundation, and that Drs. Martin, Templeton, Ross, Staunton, and Bell, be a committee to memorialize the Legislature on this subject.

*Resolved*, That the Trustees of the Medical College of Ohio, have acted unwisely in abolishing the chair of the Institutes, and that that chair ought never to be dispensed with in a well regulated medical school.

*Resolved*, That the power of appointing and removing Professors ought to be vested in a Board of Regents, who should reside in different parts of the state; and that a part of them should belong to the medical profession.

*Resolved*, That our professional brethren throughout the State, be invited to co-operate with us, in our efforts to elevate the Character of the Medical College of Ohio.

*Resolved*, That editors of newspapers throughout the State, be, and they are hereby respectfully solicited to publish these proceedings.

By order of the Society.

J. MARTIN, *President.*
J. S. PERKINS, *Secretary.*

*Waynesville, Ohio, May* 29, 1832.

### III. *Memorial of said Society to the General Assembly.*

TO THE HONORABLE, THE LEGISLATURE OF THE STATE OF OHIO:

Your memorialists, a committee appointed by the Third District Medical Society, to inquire into the condition and promote the improvement of the *Medical College of Ohio*, would respectfully represent to your honorable body—That the Medical College was incorporated by your predecessors at the session of 1818–19, and that the charter has been subsequently amended, and the institution munificently endowed. With the most eligible site in the extended Valley of the Mississippi, and the liberal patronage of the state, the College has not attained that degree of eminence, usefulness, and respectability, which was anticipated by the state, and the medical profession, at its foundation. It has existed for more than twelve years in an unprosperous condition, with small classes of pupils annually, many of whom were beneficiaries; whilst a rival institution, of nearly the same age, in an adjoining state, with an ineligible site, and with less pecuniary patronage from the state, has, by the enlarged and liberal policy of those who govern it, become eminently distinguished as a school of medicine; alluring, annually, to its halls, large classes of pupils. Your memorialists would respectfully suggest to your honorable body, the propriety and urgent necessity of the most vigilant and scrutinizing investigation into the causes of this great disparity between the two institutions.

The languishing condition of the Medical College of Ohio, which had, as your memorialists are informed, but 62 pupils at the close of last month, while that of Kentucky had 218, must be owing to defects both in organization and government. Of the present Board of Trustees but one is a Physician, and consequently they are not the best judges of the talents and qualifications of medical men, as professors. They all, except one, reside in the City of Cincinnati, and seem to look upon the Medical College, as having been instituted for the exclusive use and benefit of that city.

Professors, with but slender qualifications, have been selected for the College, from among the personal friends, family physicians, and relatives of the Trustees, without much regard to the interests of the state, or the science of medicine.

Although recent events have forced upon the Board a few men of distinguished talents and reputation, yet the pertinacity with which they have subsequently adhered to an undistinguished and foreign favorite (even though a year be required for preparation, and an important professorship be abolished) evinces but too palpably their disregard of the true interests of the institution.

With these facts before them, your memorialists feel constrained to ask your honorable body, to re-organize the Medical College; and with due deference, beg leave to suggest to your honorable body, the propriety and expediency of confiding the prerogative of appointing and removing medical Professors in the College, to a Board, selected from different sections of the state, a majority of whom should be physicians, or that your honorable body would so augment the number of Trustees, as to give a preponderating influence to those who reside without the city.

Your memorialists beg leave to refer your honorable body to the subjoined documents, trusting that the College may find in your wisdom, an emancipation from that system of favoritism and bad government which has ever blighted its fairest prospects.

JOSHUA MARTIN,
JOSEPH TEMPLETON,
JOHN ROSS,
JOSEPH STANTON,
WILLIAM BELL, *Committee.*

XENIA, Ohio, Dec. 6, 1832.

IV. ***Memorial and Resolutions of the General Medical Society of Ohio.***

At a meeting of the general medical society, of the state of Ohio, held at Columbus, January 9th, 1833, the following report and memorial were ordered to be presented to the General Assembly:

The committee appointed to enquire into "the uses and abuses of the present law, regulating the practice of physic and surgery, also, whether any law is necessary, and if the present law is deficient or injurious, what would be the general features of a law, that would be salutary in its operations," respectfully report, that they have given the subject all the consideration, which the shortness of the time would permit, and submit the following, as a brief exposition of their views on this interesting subject.

The first difficulty that presents itself in the present law, is the great number of tribunals it has created, to judge of the qualifications necessary for the admission of members. This, in itself, is a great evil, as it is impossible, to create twenty separate tribunals, of final and exclusive jurisdiction, that will be nearly alike in their estimate of the attainments necessary for a candidate to possess. These inconveniences were palpably felt from the commencement of the operation of this law, and attempted to be remedied by this society, at its first meeting, as well as the nature of circumstances would permit, by the adoption of their "Uniform rules and regulations for the government of district medical societies. Some of the district societies, however, where the manufacturing of licentiates had become a business of importance, remonstrated at the next meeting. Being unsuccessful in this society they however entrenched themselves, within the battlements of nullification. Protested that the state medical society could exercise no legitimate authority over them, and have acted as they pleased in this matter ever since.

In consequence of this state of affairs we have numerous examples of applicants for a license, being rejected by the censors of one medical district society, and admitted by those of another.

If a medical student is considered that he ought not to be admitted to an examination, either in consequence of moral dereliction, deficiency of time specified by law, or other disqualifications, he has only to make a visit to some other district, when either their sympathy for him as a stranger, or for his ten dollars, is sure to obtain for him, the most respectful attention; seldom indeed, or never, under such circumstances, has their hospitality failed to send the stranger home to his own district, armed with all the paraphanalia of a license, which legally authorises its bearer to practice physic and surgery, *any where in the state of Ohio.*

Thus while the petulence and selfishness of individual societies contravene nearly all the good effects which were contemplated by the law, its operation on the great body of the profession is onerous in the extreme, compelling them all to neglect their professional duties, and attend the meetings of the district society, or pay fines for non attendance, while they may be, and frequently are, taxed for the maintainaece of said societies. They must also pay tax for the support of the state medical society. Which taxes, in conjunction with the professional tax, they have to pay the state, in the same manner as attorneys, increases their tax expenditures almost beyond endurance. While the empyric is exempt from all taxation and inconvenience, and liable to no penalty let him sport with life and health ever so wantonly. Nine years of patient observation, and painful experience, have compelled us to think, that the present law ought to be repealed.

As our opinion is required by the resolution under which this report is made, whether any law would be salutary in its operations? We answer in the affirmative, for the following reasons. As nations of the earth emerge from barbarism, and advance in civilization and refinement, as the arts and sciences become generally diffused among mankind, and ignorance and superstition subside, in the same proportion are the health and lives of the community more highly estimated. Those nations of the earth which have excelled in the arts, both of peace and war, have been and still are the foremost by wise Legislative enactments, to protect their people from ignorance, superstition and imposture in the healing art.

Nevertheless, mingled with the most intelligent communities, will be found too many whose grade of intelligence will steadily permit them, to become the dupes of the pretender to medical skill, who conscious of his ignorance, will exert his more available talents of deception, intrigue, and flattery, to ingratiate himself into the good graces of the honest and worthy, but ignorant and unsuspecting citizen—consequently the propriety, indeed the moral necessity, of legislative provision, not for the benefit of the profession, properly so called, but for the protection of the people. In the expression of this opinion we are governed by no sinister motives. Were selfishness our object we would be silent. The medical profession have no common consolidated pecuniary interest, but quite the reverse. Their interests are so insolated and conflicting in relation to professional emoluments and fame, that nothing less than the active benevolence, and deep toned moral feeling for which the enlightened of the profession are every where proverbial, could induce them to be thus frank in the expression of sentiments, so generally opposed by popular prejudices. A law that would really promote the usefulness of the profession, and render general satisfaction, must be simple in its application, and plain in its provisions. One board of examiners for the whole state would be much preferable to a plurality, and provision ought to have been made that these examiners be selected by proper authorities for their moral virtues, professional skill, and scientific attainments. The time of their meeting, their duties and powers, ought to be specified; while the illegal and presumptuous practitioner ought to be made subject to a competent fine of penalty; making it the duty of the grand jury to find a bill, and of the attorney for the commonwealth to prosecute, as in other cases of crime and misdemeanors.

Deeply connected with the true interests of the community, as well as with the dignity of the profession, is the situation and prospects of the Medical College of Ohio. In consequence of the liberality extended to it by the Legislature, and the growing intelligence of the people of this great state, we confidently hope the time will soon arrive, when no honest young man will presume to take upon himself the awful responsibilities of the medical profession, unless he carries with him the authority of it, or some other respectable medical school. But it is with pain we must confess, that we have seen the professorship of the Institutes of the medicine abolished in that institution. A professorship of the utmost importance in a well regulated medical school and essential to the student when filled by a professor of competent talents.

We would humbly hint to the Legislature, that the cause of humanity and science, would seem to require their most scrutinizing and anxious attention to this College, as the best and most effectual means of directing public opinion on the subject of medical qualifications.

In the mean time if the present law is repealed, a law might be passed regulating the practice of medicine, embracing the provisions heretofore intimated, for the purpose of protecting the community, while at the same time it would confer upon them, all the advantages that could be derived from the labours of such young men, as from pecuniary embarrassments, could not obtain a diploma, but whose talents, acquirements, and industry, would render them safe practitioners and useful members of society.

In conformity with these views, hastily sketched, but we hope not the less true in substance, we would offer for the consideration of the society, the following resolutions:

Resolved, That the present law regulating the practice of physic and surgery has failed to affect the object for which it was designed, and therefore ought to be repealed.

Resolved, That the attention of the Legislature be specially invited to the medical College of Ohio, as the most effectual means of properly directing public opinion in relation to medical qualifications.

### MEMORIAL.

*To the Honorable the Senate and House of Representatives, in General Assembly convened.*

Your memorialists, the Ohio State Medical Society, now convened, would respectfully represent to your honorable body, that for more than ten years, the attention of the medical profession in this state has been fixed upon the medical College of Ohio, with the fond expectation that endowed with the liberal patronage of the state, possessing the most eligible situation to be found in the western country, it would attain a degree of eminence and usefulness, not inferior to any medical school in our country. That the intelligent and enterprizing medical students, of our own state, would here find an institution, where they might attain the highest honors of their profession; and not be compelled to attend the Lectures of Professors of Eastern schools, who must necessarily be in some degree unacquainted with the character of our western diseases, or to attend a neighboring rival institution.

Your memorialists are constrained to declare, that in their opinion the present board of Trustees have acted without due regard to the interests of the institution in abolishing the chair of the institutes, inasmuch the institutes of medicine is the very foundation of medical education upon which the whole superstructure depends.

Your memorialists believe that the concerns of the institution have been so managed as to give general dissatisfaction to the profession, and to diminish their confidence in its operations and usefulness; and humbly pray your honorable body, to so change and extend the board of Trustees, as that it may hereafter be governed upon such principles as shall conciliate confidence and patronage, extend its usefulness, and elevate its reputation. And do heartily concur with the memorialists on the same subject, of the third medical district, in suggesting to your honorable body the propriety and expediency of confiding the prerogative of appointing and removing medical professors in the college, to a board of Regents, selected from different sections of the State.

And your memorialists as in duty bound will ever pray, &c.

By order of the Society,

T. FLANNER, *President.*

R. M'NEILL, *Secretary.*

In pursuance of these memorials, the General Assembly repealed the law regulating the practice of physic, and consevuently abolished the District Secioties and the General Medical Society, composed of a representative from each district.

V. Resolution appointing a Board of Commissioners to inquire into the condition of the Medical College of Ohio, and the Commercial Hospital and Lunatic Asylum of Ohio.

" *Resolved by the General Assembly of the State of Ohio,* That the Governor of this State, be, and he is hereby authorized and requested to appoint five citizens of this State (not connected with the institutions herein mentioned,) at least three of whom shall be physicians, to visit, on the second Monday in April next, the Medical College of Ohio at Cincinnati, and to enquire into and examine the present organization, government and condition of said Medical College, and the arrangements of the Trustees of said Institution, for the instructions of pupils and the advancement of Medical Science therein; and also to enquire into the condition, government and arrangements for the attaining the purposes thereof of the Commercial Hospital and Lunatic Asylum, and that they report thereupon to the next General Assembly by the third Monday of December next; with such observations and suggestions in relation to the said institutions, and the proper means of advancing the prosperity and utility of said Medical College as an institution of the State, and of Medical Science therein, and of the said Hospital and Asylum as connected therewith, as they may think proper to make. The persons thus appointed to receive from the Governor's contingent fund or otherwise a reasonable compensation for the time severally employed by them in the service provided by this Resolution, and the said Commissioners shall have power to send for persons and papers and to take testimony under oath.

*Resolved further,* That so much of the result of the examinations as aforesaid as concerns the public confidence in those institutions, as in the opinions of the aforesaid Committee would be proper to be published, the Committee shall cause to be published in two or more of the newspapers having general circulation in the State."

In obedience to the Resolution, the Governor appointed the following gentlemen as Commissioners:

JOHN W. WILLEY, Esq. of Cuyahoga County.
Dr. JOHN M. EDMISTON, of Franklin.
Dr. M. Z. KREIDER, of Fairfield.
Dr. ROBERT THOMPSON, of Guernsey.
Dr. WILLIAM S. KITTRIDGE, of Huron.

The dissensions prevailing among the Physicians of this city, growing out of the Medical College of Ohio, are well known to the profession throughout the Western Country. These disputes have done any thing rather than advance the respectability of the profession, or the interests of medical science. With a view of putting an end to so unpleasant a state of things, the Legislature has appointed a Board of Commissioners with full power to inquire into the cause of the existing dffiiculties, who will report the result of their investigations to the next General Assembly.

We have published the documents which have led to this investigation in order that the profession in the West may have a full view of the causes of complaint against the present organization of the Medical College. The principal charges against the Board appear to be—1st. A spirit of favoritism, necessarily, perhaps, resulting from the influence which medical men must always exert over the society in which they live, from the nature of the relation in which they stand towards it. 2ndly. A want of qualification from the absence of medical men in the Board.

The remedy proposed is, a Board of Regents, and who shall be, by their locality, beyond the local influence of Cincinnati, embracing a proportionate number of medical men.

One advantage at least, we hope, will result from the investigation: which is, that some permanent arrangement will be made, which, by precluding the prospect of further change, will put an end to all future dissension.

